# Supplementary material for: Loss of Extrasynaptic Inhibitory Glycine Receptors in the Hippocampus of an AD Mouse Model Is Restored by Treatment with Artesunate
Source: Int J Mol Sci. 2023 Feb 27;24(5):4623. doi: 10.3390/ijms24054623 (PMC10002537; doi:10.3390/ijms24054623)
Supplement: Supplementary file 1 [file ijms-24-04623-s001.zip › ijms-2141304-supplementary.pdf]

Supplementary Figure S1

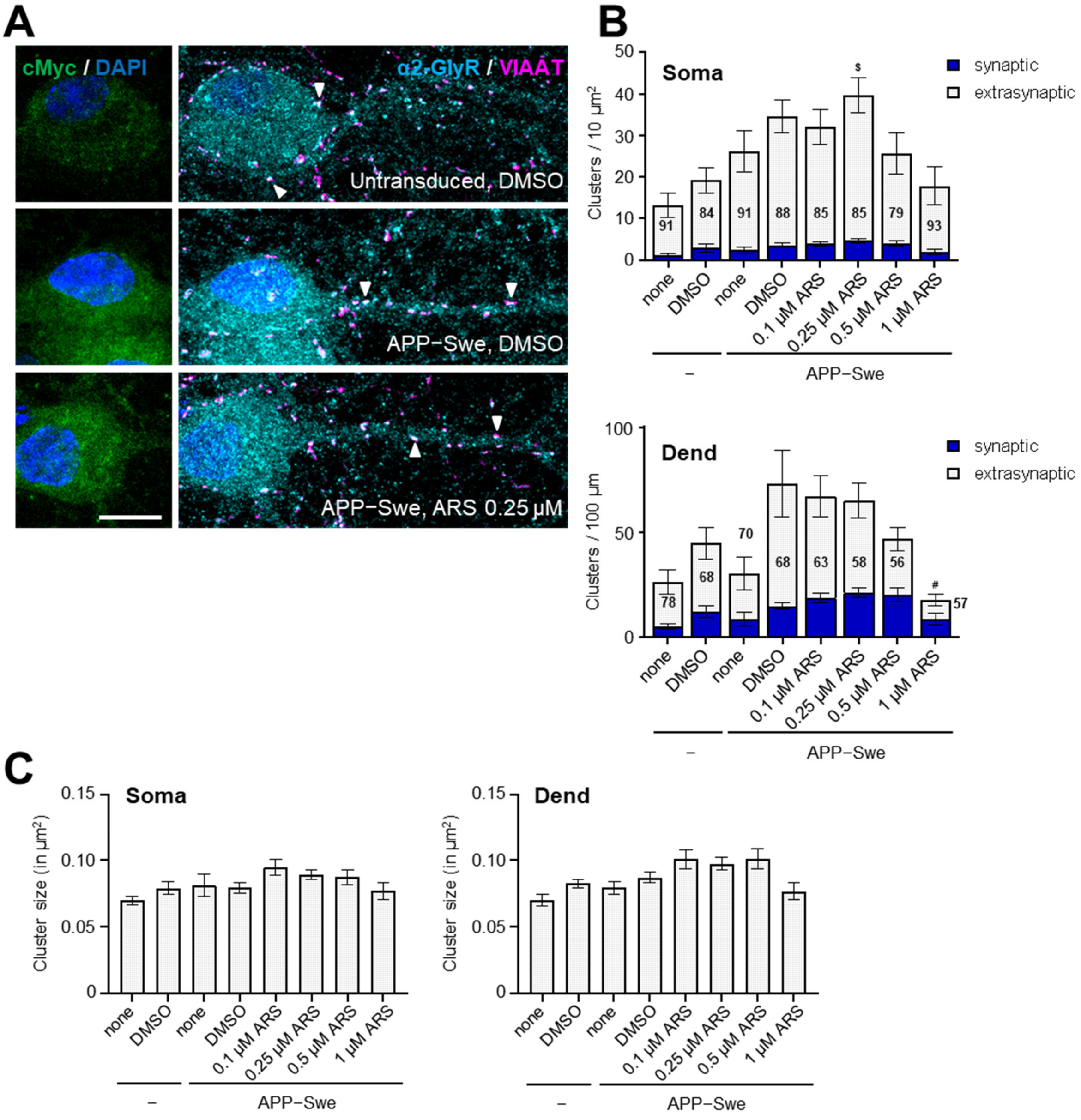

Legend Supplementary Figure S1. The effects of APP-Swe overexpression and artesunate treatment on alpha2-containing GlyR clusters in dissociated hippocampal neurons. (**A**) Representative IF images of transduced hippocampal neurons of selected groups. cMyc tagged APP-Swe transduced cells were manually selected for analysis based on cMyc immunofluorescence. Regions of interest encompassing either the entire soma or the proximal dendrite were selected and analyzed. The presence of clear synaptic (overlapping with presynaptic

VIAAT signal) and extrasynaptic alpha2-containing GlyR clusters (no overlap) can be clearly seen, as well as a diffuse cytoplasmatic staining. (**B**) Quantification of the density of alpha2-GlyR clusters at both the somatic (upper graph) and proximal dendritic (lower graph) compartments. Clusters were subdivided in synaptic (blue bars) and extrasynaptic (light grey bars) clusters based on their overlap with VIAAT signal. The numbers represent the percentage of extrasynaptic clusters. Cluster density varied between the different treatment groups, a statistically significant increase of extrasynaptic alpha2-GlyR clusters was found only for the 0,25 μM artesunate treatment in the somatic compartment and a decrease for the 1 μM artesunate treatment in the dendritic compartment. (**C**) Quantitative analysis of cluster size showed no effects of either APP-Swe overexpression or artesunate treatment. Results depicted as mean ± SEM. n = cells (20-24 per group). # signifies a statistically signifant change ($p < 0.05$) compared to APP-Swe-DMSO, $ signifies a statistically significant change compared to untransduced-DMSO (One-way ANOVA with Sidak's multiple comparison tests).
